# Synchronous Hodgkin's lymphoma and seminoma: a rare coexistence and an important lesson

**DOI:** 10.1002/ccr3.1140

**Published:** 2017-08-30

**Authors:** Prabhsimranjot Singh, Sudhamshi Toom, Makhardwaj Shrivastava, Jason Shaw, David Silver, Jasminka Balderacchi, Jay Lipshitz

**Affiliations:** ^1^ Department of Hematology/Oncology Maimonides Medical Center Brooklyn New York; ^2^ Department of Internal Medicine Maimonides Medical Center Brooklyn New York; ^3^ Department of Thoracic surgery Maimonides Medical Center Brooklyn New York; ^4^ Department of Urology Maimonides Medical Center Brooklyn New York; ^5^ Department of Pathology Maimonides Medical Center Brooklyn New York

**Keywords:** Germ cell tumor, Hodgkin's lymphoma, mediastinal lymphadenopathy, seminoma

## Abstract

Synchronous presentation of seminoma and lymphoma is rare but has important ramifications for the treatment of both malignancies. Without clinical vigilance, this situation may be easily missed, leading to inappropriate management. We describe a patient with synchronous seminoma and Hodgkin's lymphoma and discuss the implication on his treatment.

## Case Presentation

**Figure 1:**
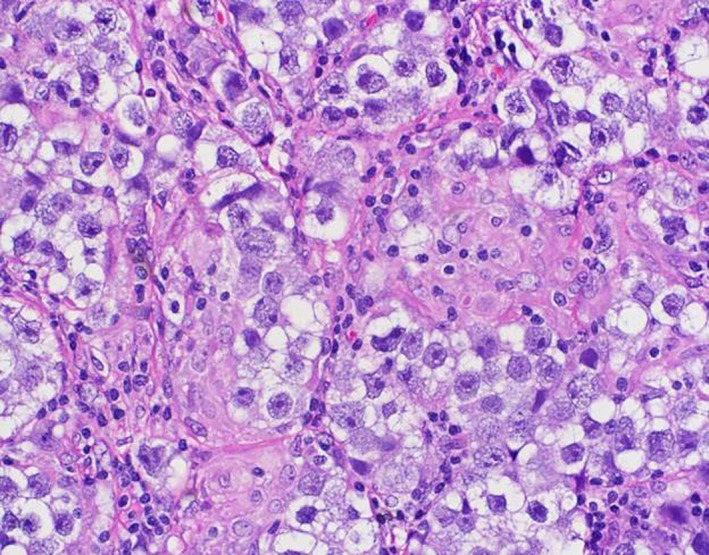
Testicular mass—Seminoma.

**Figure 2:**
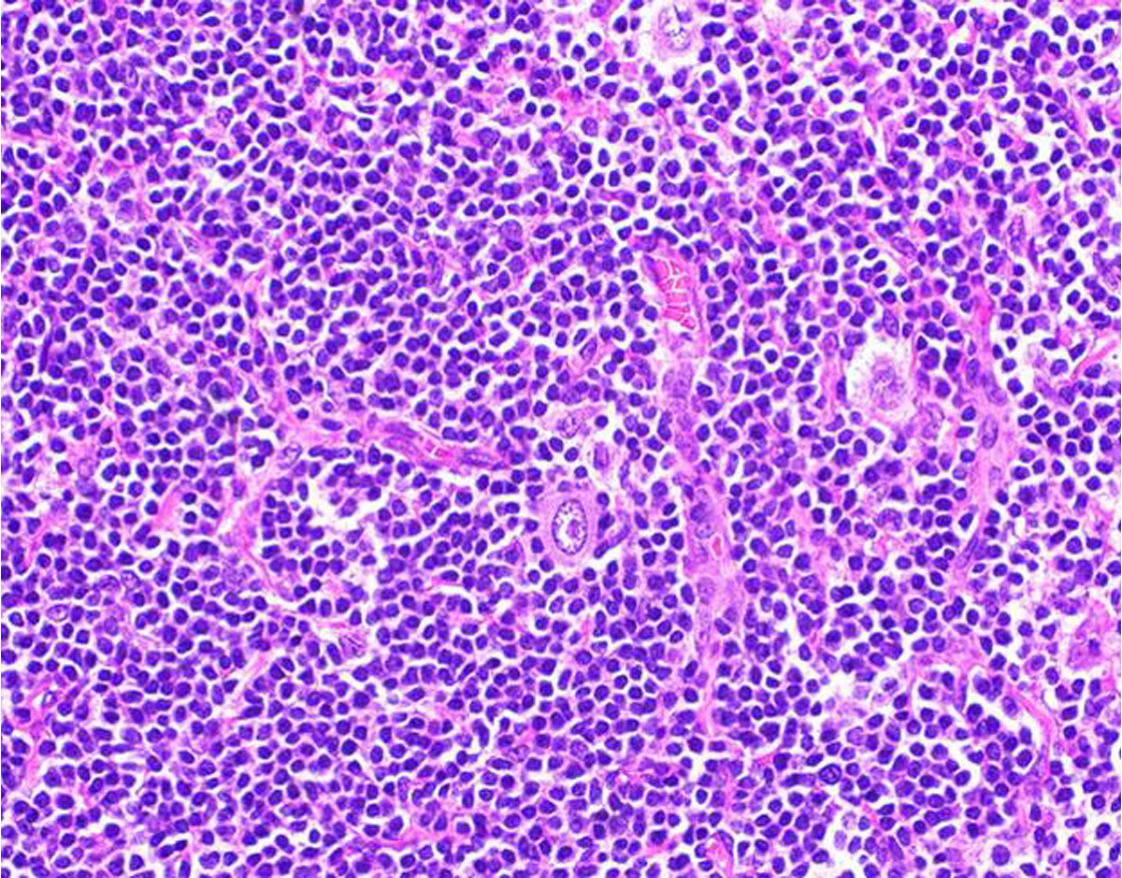
Lymph node biopsy—Classic Hodgkin's lymphoma.

A 59‐year‐old man with no significant medical history presented with progressive swelling and erythema of the right testis. Testicular cancer was suspected and he underwent a radical right inguinal orchiectomy. Pathology revealed a 5.7 cm seminoma of the testis with lymphovascular invasion and without spermatic cord involvement (pT2) (Fig. 1). His tumor markers including AFP, LDH, and Beta‐HCG were normal (S0). A CT scan of the chest, abdomen, and pelvis followed by a PET/CT revealed enlarged, hypermetabolic mediastinal, hilar, and periportal lymphadenopathy interpreted by the radiologist as concerning for metastatic disease. Given the atypical distribution for lymphadenopathy from testicular seminoma, an excisional biopsy of a left hilar node was performed and revealed Classical Hodgkin's lymphoma with IHC positive for CD15, CD30, and PAX‐5 (Fig 2). He denied any B‐symptoms and his bone marrow was uninvolved by lymphoma (stage IIIA). Adjuvant therapy for his germ cell tumor, otherwise an important consideration, was deferred, and he began chemotherapy with adriamycin, bleomycin, vinblastine, and dacarbazine (ABVD) for six cycles. Interim PET/CT after two cycles of ABVD showed a complete response. He has completed six cycles of ABVD and chose observation as opposed to single dose of adjuvant carboplatin for his seminoma and is currently under surveillance for both malignancies.

## Discussion

The unusual coexistence of Hodgkin's lymphoma and seminoma has rarely been documented in medical literature, with three other cases previously reported 1, 2. In each case, a biopsy of lymphadenopathy, primarily outside the retroperitoneum, yielded a diagnosis of lymphoma. Both Hodgkin's lymphoma and germ cell tumors commonly involve lymph nodes and present in young men. Lymphadenopathy may understandably be assumed to represent metastatic disease in a young man with known testicular cancer. Clinical vigilance is necessary to question the nature of atypical sites of lymphadenopathy in such a patient, and to pursue the possibility of an alternate diagnosis with a lymph node biopsy. A missed diagnosis of lymphoma in such a patient would also mean harmful overstaging of the germ cell tumor. These two cancers represent two of the most curable malignancies. Oncologists must therefore be extra vigilant in approaching these patients to accurately diagnose and stage them, to be able to institute appropriate therapy.

## Conclusion

Our report highlights the importance of clinical suspicion of a lymphoma in patients with another cancer and lymphadenopathy not typical of metastatic disease for that tumor type. In such situations, a lymph node biopsy is crucial to proceed with the correct therapy for each malignancy. While the simultaneous presentation of Hodgkin's lymphoma and seminoma is rare, cases like ours highlight the importance of questioning metastatic disease when lymphoma seems to be a possibility.

## Authorship

PS: collected data, wrote, and critically analyzed the manuscript. ST: collected data, and wrote the manuscript. MS: collected data and analyzed the manuscript. JS: critically analyzed the manuscript. DS: critically analyzed the manuscript. JB: critically analyzed the manuscript. JL: collected data and critically analyzed the manuscript.

## Conflict of Interest

None declared.
